# Transplantation of Menstrual Blood-Derived Mesenchymal Stem Cells Promotes the Repair of LPS-Induced Acute Lung Injury

**DOI:** 10.3390/ijms18040689

**Published:** 2017-03-27

**Authors:** Bingyu Xiang, Lu Chen, Xiaojun Wang, Yongjia Zhao, Yanling Wang, Charlie Xiang

**Affiliations:** State Key Laboratory for Diagnosis and Treatment of Infectious Diseases, Collaborative Innovation Center for Diagnosis and Treatment of Infectious Diseases, The First Affiliated Hospital, College of Medicine, Zhejiang University, Hangzhou 310027, China; jqdxxby@126.com (B.X.); chenlu20602@163.com (L.C.); wangxiaojun1637@126.com (X.W.); liebeqiu@126.com (Y.Z.); wangyanling@zju.edu.cn (Y.W.)

**Keywords:** ALI, mesenchymal stromal/stem cells, MSC treatment

## Abstract

Acute lung injury (ALI) and acute respiratory distress syndrome (ARDS) are associated with high morbidity and mortality. Menstrual blood-derived stem cells (MenSCs) have been shown to be good therapeutic tools in diseases such as ovarian failure and cardiac fibrosis. However, relevant studies of MenSCs in ALI have not yet proceeded. We hypothesized that MenSC could attenuate the inflammation in lipopolysaccharide (LPS)-induced ALI and promote the repair of damaged lung. ALI model was induced by LPS in C57 mice, and saline or MenSCs were administered via tail vein after four hours. The MenSCs were subsequently detected in the lungs by a live imaging system. The MenSCs not only improved pulmonary microvascular permeability and attenuated histopathological damage, but also mediated the downregulation of IL-1β and the upregulation of IL-10 in bronchoalveolar lavage fluid (BALF) and the damaged lung. Immunohistochemistry revealed the increased expression of proliferating cell nuclear antigen (PCNA) and the reduced expression of caspase-3 indicating the beneficial effect of MenSCs. Keratinocyte growth factor (KGF) was also upregulated after MenSCs administrated. As shown using transwell co-culture, the MenSCs also could improve the viability of BEAS-2B cells and inhibit LPS-induced apoptosis. These findings suggest that MenSC-based therapies could be promising strategies for treating ALI.

## 1. Introduction

Acute lung injury (ALI) and its severe form, acute respiratory distress syndrome (ARDS), is a multifactorial syndrome characterized by an acute onset, increased lung permeability, intractable hypoxemia and the absence of cardiogenic pulmonary edema [1,2,3]. Despite decades of intensive research efforts, there are still no effective therapies for the disease [4] and the mortality rate ranges from 22–40% [3,5]. Safer and more effective therapies are urgently needed.

One area of interest is the potential for mesenchymal stem cells (MSCs) to be used for repairing the injured lung tissue. MSCs were first referred to as colony-forming unit fibroblasts upon their discovery by Friedenstein [6] and possess the capacity for self-renewal, multipotency and cytokine secretion. These characteristics make them ideal for tissue repair or regeneration applications [7,8,9]. Recent studies suggest that stem/progenitor cells contribute to lung repair when they are used as cellular therapies [10,11,12,13,14,15]. MSCs not only can migrate to the injured lung tissue, thereby enhancing the therapeutic effect, but also attenuate the injury through paracrine functions [16,17]. However, the MSCs such as Embryonic stem cells (ESCs) and bone marrow mesenchymal stem cell (BM-MSCs) are hampered by moral objections and ethical requirements for acquisition. This severely limits their application in research and clinic.

Menstrual blood-derived stem cells (MenSCs) are highly proliferative stem cells existed in the endometrium and were first identified by Men [18]. MenSCs not only have beneficial phenotypes and properties, including the ability of differentiate into the three germ lineages [19,20,21], but also could be expanded on a clinically relevant scale in a relatively short time without developing genetic abnormalities [18,22]. As they are isolated from human menstrual blood, MenSCs are easily accessible and would not be harmful to the human body [23,24]. Additionally, MenSCs are hypoimmunogenic due to the low expression of major histocompatibility complex class II and costimulatory molecules on their surface [23,25,26]. This characteristic renders their application more feasible, whether between the same or different species. What is more, Zhong et al. implanted MenSCs in 4 patients with multiple sclerosis revealed no immunological reactions or treatment associated adverse effects after a long follow up [27]. These make MenSCs ideal for clinical use [23,27,28] and have been used in several disease models [23,29,30,31,32,33,34]. Cui et al. found MenSCs could transfer dystrophin into dystrophied myocytes through cell fusion and transdifferentiation in vitro and in Duchenne muscular dystrophy (DMD) model mice. Jiang et al. implanted MenSCs intramyocardial into rat model of myocardial infarction (MI) and significantly preserved viable myocardium in the infarct zone and improved cardiac function. Our group also demonstrated MenSCs could improves hyperglycemia by promoting endogenous progenitor differentiation in type 1 diabetic mice. However, the application of MenSCs in ALI has not been reported. The aim of our current study was to identify the function of MenSCs in the lipopolysaccharide (LPS)-induced ALI.

## 2. Results

### 2.1. Characterization of the MenSCs

**Figure 1:**
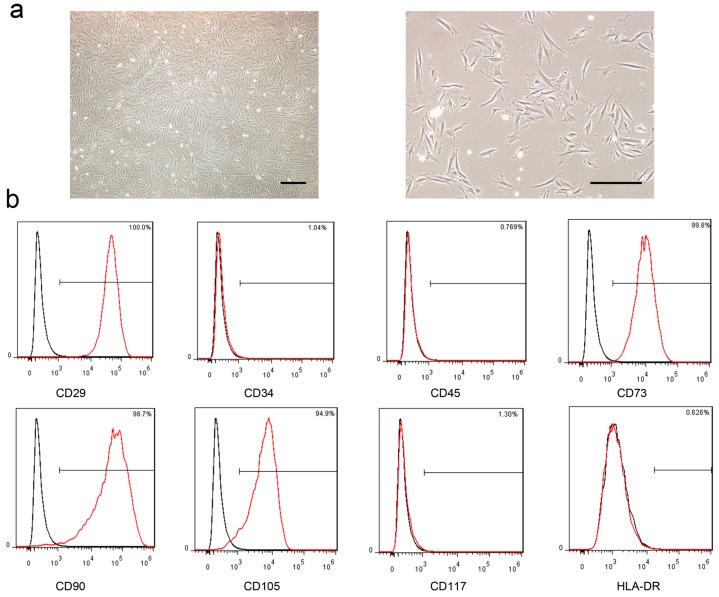
Characterization of MenSCs. (**a**) By microscopy, MenSCs exhibited a spindle-shaped, fibroblast-like morphology (200×), Scale bar: 50 µm; (**b**) Representative surface markers of MenSCs were detected by flow cytometer. The black lines represent the isotype control and the red lines represent the level of the surface markers.

As determined by microscopy, the MenSCs were adherent to the plastic tissue culture flasks and spindle-like in shape (Figure 1a). The flow cytometric analysis indicated that the MenSCs were positive for CD29, CD73, CD90, and CD105, markers of MSCs, and negative for CD34, CD45 and CD117 (Figure 1b). These results revealed that the MenSCs were not hematopoietic. The low expression of human leukocyte antigen DR (HLA-DR) indicated the low immunogenicity of the MenSCs, which would render these cells ideal for transplantation into animals or humans.

### 2.2. Migration of MenSCs In Vivo and In Vitro

**Figure 2:**
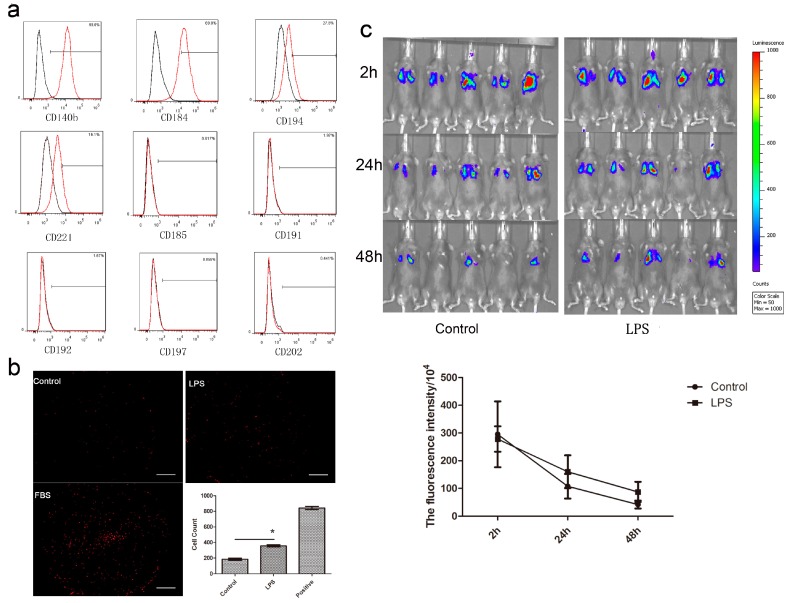
Migration of MenSCs in vitro and in vivo. (**a**) MenSC Surface markers for migration. The black lines represent the isotype control and the red lines represent the levels of the surface markers; (**b**) Migration of MenSCs assessed using the transwell system. After migration, MenSCs adhered to the underside of the membrane were stained with Dil and counted under the fluorescence microscopy. The summative histograms are shown; (**c**) Migration of MenSCs to the lung of mice. MenSCs were allowed to migrate in the mice for three days and observed using a live imaging system (**upper** panel) at 2, 24 and 48 h, after which the fluorescence intensity was measured (**lower** panel). Three independent experiments with similar results were performed. * *p* < 0.05. Scale bar: 1000 µm.

Nine receptors of chemokines or cytokines with chemotaxis were detected by flow cytometry. We found MenSCs expressed CD194, CD221, CD140b and CD184 strongly (Figure 2a), while the other markers were not expressed. These results indicated that MenSCs could be attracted by certain chemokines and have a strong migration ability. The transwell experiments demonstrated MenSC migration in vitro. In the LPS group, 17.8% of MenSCs migrated from the transwell inserts to the lower layer of the membrane, while only 9.5% of cells in the control group migrated. The difference between the two groups was significant (Figure 2b). In vivo, the migration of MenSCs was monitored for three days. On day 1, the MenSCs in both groups remained in the lungs of the mice and displayed a strong signal. Over the next two days, both of the signals of the control group and ALI group decreased. Although statistically not significant, LPS-treated group showed the retention of more cells in the lung (Figure 2c). These results demonstrated that MenSCs could migrate to and be retained in the injured area to potentially serve an important function in treating ALI.

### 2.3. MenSCs Protect BEAS-2B Cells from LPS Injury

**Figure 3:**
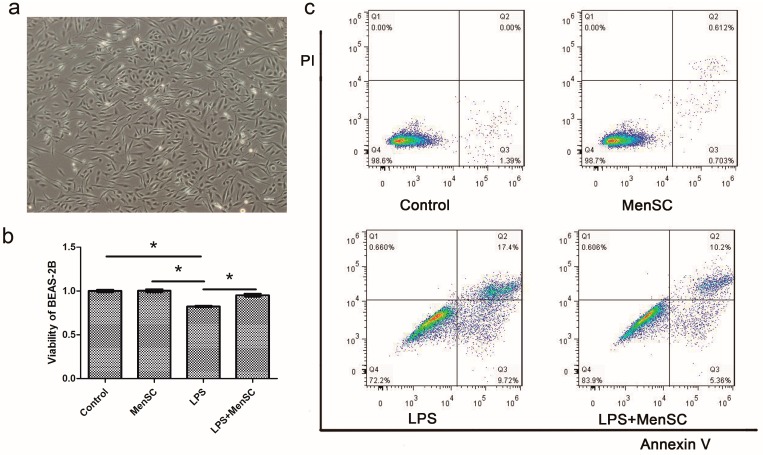
MenSCs protect BEAS-2B cells from LPS injury. (**a**) Morphology of BEAS-2B cells observed by microscopy; (**b**) Viability of BEAS-2B cells in four groups after co-cultured with or without LPS and MenSCs; (**c**) Apoptosis of BEAS-2B cells after co-culture was assessed by Annexin V/PI. Numbers represent the proportion of BEAS-2B in the restricted image. The experiment was repeated triple. PI: Propidium. Iodide * *p* < 0.05. Scale bar: 50 µm.

To determine the effect of MenSCs on BEAS-2B cells (Figure 3a), i.e., human lung epithelial cells, we assessed cell viability and apoptosis after 24 h of co-culture. BEAS-2B cells exposed to LPS exhibited a significant reduction in viability compared to the normal BEAS-2B cells, and MenSCs reversed this reduction (Figure 3b). Similar results were observed for BEAS-2B cell apoptosis. Compared with the normal BEAS-2B cells, the cells exposed only to LPS had a significantly higher apoptosis rate, and the MenSCs could protect the cells from LPS injury (Figure 3c).

### 2.4. Differentiation of MenSCs into Lung Epithelial Cells

**Figure 4:**
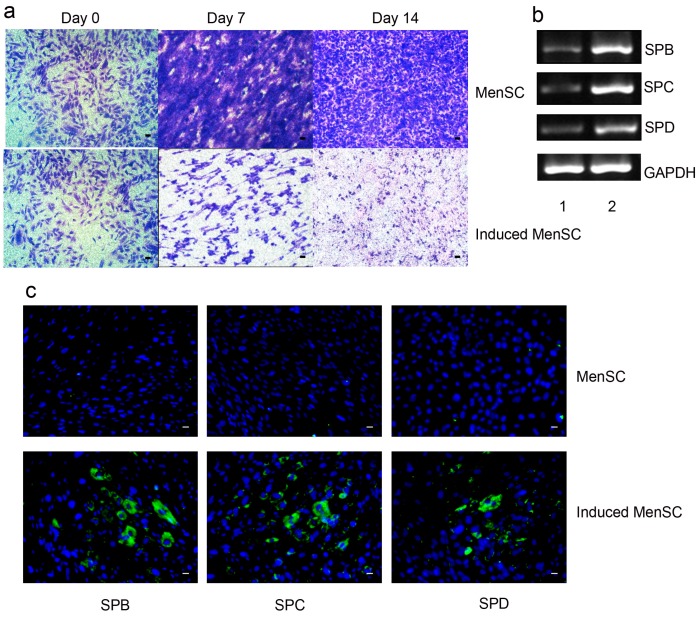
Differentiation of MenSCs into lung epithelial cells. (**a**) The shape of the cells had changed from spindle-like to polygonal-like, similar to that of lung epithelial cells; (**b**) After induction, MenSCs expressed SPB, SPC and SPD, markers of lung epithelial cells while the control group remains none; (**c**) RT-PCR confirmed the expression of MenSC markers before (lane 1) and after (2) induction; Scale bar: 50 µm.

After two weeks of induction, the MenSCs were observed by light microscopy. The shape of the cells had changed from spindle-like to polygonal-like, similar to that of lung epithelial cells (Figure 4a). Immunofluorescence staining indicated that after induction, most MenSCs expressed SPB, SPC and SPD, markers of lung epithelial cells (Figure 4b). The RT-PCR analysis confirmed these results (Figure 4c). After induction, the expression of SPB, SPC and SPD in MenSCs were regulated. These findings indicated that the MenSCs were not only capable of differentiating but could also be induced to differentiate into lung epithelial cells.

### 2.5. MenSCs Relieve Symptoms of ALI

**Figure 5:**
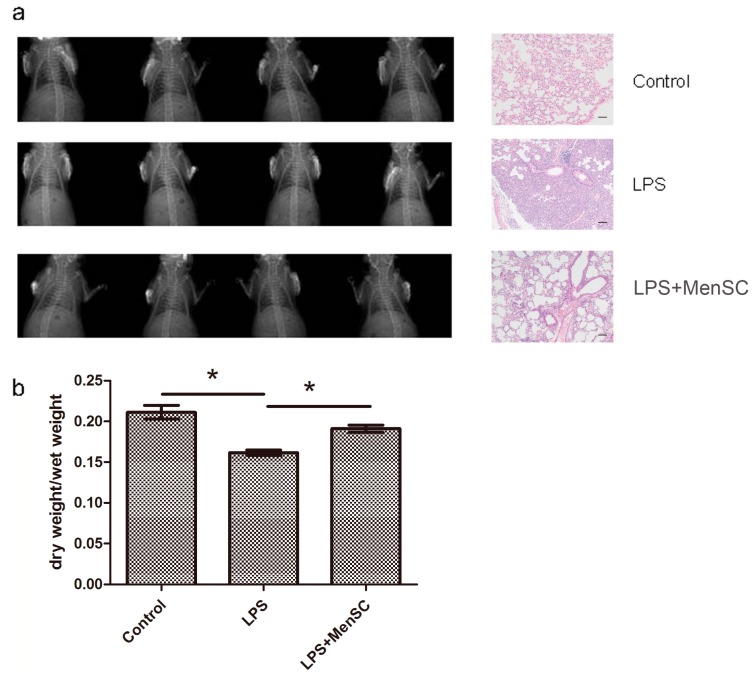
MenSCs relieve symptoms of ALI. (**a**) Representative Lung X-rays (**left** panel) and pathological variances (H&E staining, **right** panel) of different groups. The lung of control group showed no obvious lesion in the lung tissue. The lung of LPS group exhibited obvious injury symptoms of inflammation, interstitial edema and hemorrhage, which were attenuated after MenSC transplantation (LPS + MenSCs group); (**b**) Lung dry/wet ratios were analyzed by one-way ANOVA. * *p* < 0.05. Scale bar: 50 µm.

Inflammation is a hallmark of ALI. Serious lung inflammation and edema occurred after the intratracheal administration of LPS. The X-rays revealed that the lung texture thickened, and the dry/wet ratio decreased from 0.212 to 0.161 (*p* < 0.05). Treatment with MenSCs attenuated the observed inflammation, mitigating the thickened texture and increasing the dry/wet ratio (Figure 5a,b). Hematoxylin and Eosin (H&E) staining of the lung samples also confirmed the phenomenon. In the LPS group, the interalveolar septa were thickened, and the alveoli were filled with inflammatory cells, indicating extensive morphological damage compared to the control group. When MenSCs were administered, the injury was reversed. These results demonstrated that the lung histopathology had improved. (Figure 5a).

### 2.6. MenSC Attenuate the Inflammation of ALI

**Figure 6:**
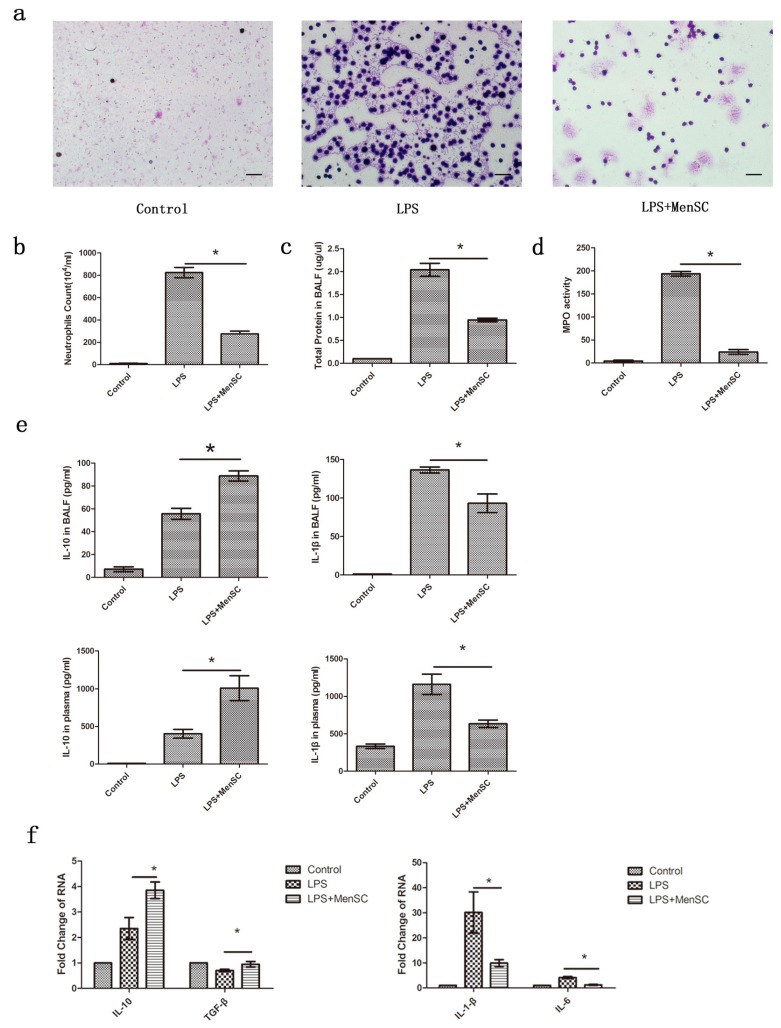
MenSCs attenuate the inflammation of ALI. (**a**,**b**) inflammatory cells in the BALF were observed by cell smears (Wright-Giemsa staining) and counts; (**c**) The total protein in the BALF; (**d**) MPO activity in the BALF; (**e**) IL-10 and IL-1β protein levels in the BALF and plasma; (**f**) Expression levels of IL-10, TGF-β, IL-1β and IL-6 in the lung. * *p* < 0.05. Scale bar: 50 µm.

BALF changes are important markers for evaluating lung function. From the cell smears, we found that the BALF inflammatory cell count was much lower in the MenSC group than in the LPS group (Figure 6a,b). Similarly, the total protein in the BALF and the MPO activity were increased in the LPS group and reduced in the MenSC group (*p* < 0.05) (Figure 6c,d). The same trends were observed for the inflammatory cytokine levels in the BALF. At 48 h after LPS was administered, the levels of IL-10 and IL-1β were 55.63 and 136.24 pg/mL, respectively. When MenSCs were administered, the level of IL-10 increased to 88.78 pg/mL, while the level of IL-1β decreased to 93 pg/mL, indicating attenuated inflammation (Figure 6e). Additionally, the plasma concentrations of IL-10 and IL-1β exhibited a similar trend.

Real-time PCR also demonstrated the function of MenSCs. We detected the gene expression of the pro-inflammatory cytokines IL-1β and IL-6 as well as the anti-inflammatory cytokines IL-10 and TGF-β. The expression of IL-10 and TGF-β were reduced in the lungs of the LPS group and were significantly elevated after the MenSC treatment. Similarly, the levels of IL-1β and IL-6 were higher in the LPS group than in the control and MenSC groups (Figure 6f). These results suggest that MenSCs may attenuate the inflammatory response via regulating the expression of cytokine.

### 2.7. MenSCs Promote the Repair of Damaged Lung Tissue

**Figure 7:**
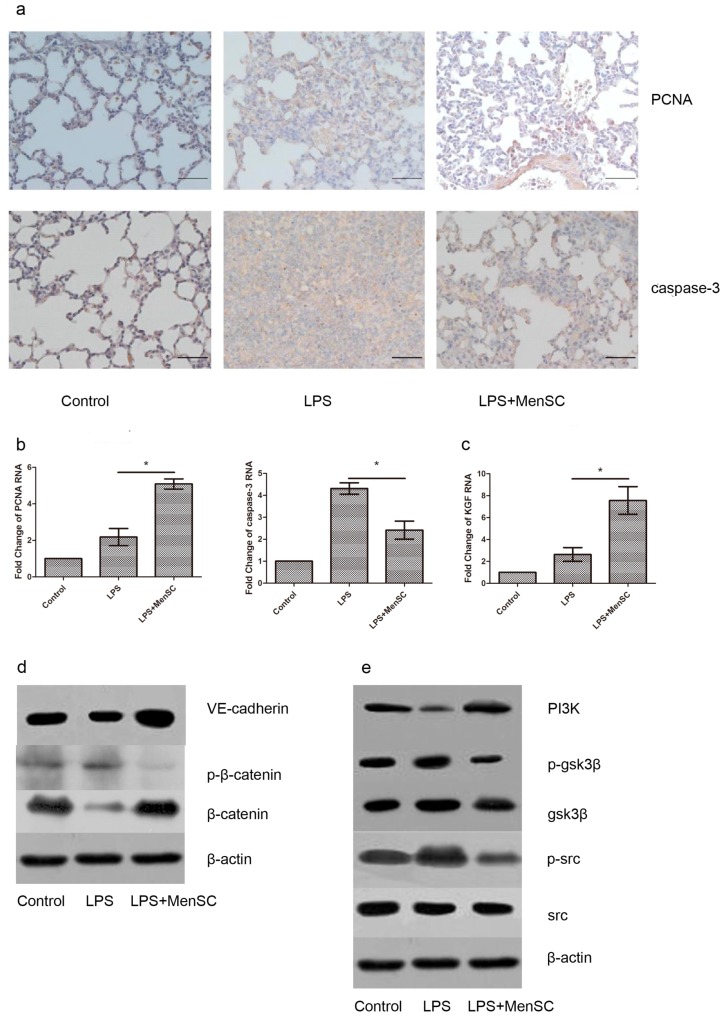
MenSCs promote the repair of damaged lung tissue. (**a**) Representative immunohistochemistry images for PCNA and caspase-3 of different groups; (**b**) Expression levels of PCNA and caspase-3 in the lung tissue; (**c**) KGF expression level in the lung tissue; (**d**,**e**) Western blot analysis of protein levels of VE-cadherin, β-catenin, p-β-catenin, Src, p-Src, PI3K, gsk3β, p-gsk3β and β-actin in the lung tissues of different groups * *p* < 0.05. Scale bar: 50 µm.

PCNA was a good index to estimate the proliferative activity [35] and caspase-3 was an important index of apoptosis [12]. Immunochemistry and RT-PCR revealed that the expression of PCNA was increased in the MenSC group compared to the LPS group, while the expression of caspase-3 was significantly attenuated (Figure 7a,b). Blinded evaluations of the PCNA and caspase-3 immunostaining and independent observation were carried out simultaneously by two experienced pathologists. KGF, a potent mitogenic factor for alveolar epithelial cells, was also expressed to a greater extent in the MenSC group (Figure 7c). We also found the protein levels of VE-cadherin, β-catenin, PI3K were decreased in the LPS group and increased after MenSCs were implanted (Figure 7d,e). Meanwhile, we found the upregulation expressions of p-gsk3β, p-src and p-β-catenin in the LPS group and the expressions were reversed after MenSCs implanted. These results indicated MenSCs could repair the injury lungs and restored alveolar-capillary membrane function through PI3K/ β-catenin cross-talked with the gsk3β/β-catenin pathway.

## 3. Discussion

MenSCs are menchymal stem cells isolated from menstrual fluids and devoid of ethical dilemmas or medical complications for cells harvesting compared to MSCs from other sources such as placental and bone marrow. The non-invasive isolation procedure makes it possible of periodic collections from the same donor and ensures higher therapeutic doses of low passaged MSCs from the same genetic background. What is more, the growth kinetics and clonality of MenSCs were significantly higher and these cells could be propagated for longer periods in culture without detectable changes in their proliferation rate [18,36]. These characteristics make MenSCs an ideal for cell therapy.

In this study, we identified the surface markers of MenSC. They exhibited some characteristics as BM-MSC such as low expression of CD34, CD45, and hematopoietic-specific markers. However, MenSCs do not express CD117, which is a common marker of BM-MSC [37]. The low expression of HLA-DR renders their application more feasible. The advantages of MenSCs make it an ideal tool for use in the present study.

The retention of injected cells is one of the foremost advantages of cell-based therapies. In our study, we found MenSCs expressed CD194, CD221, CD140b and CD184 strongly which made MenSCs could migrate to the inflammation site when the injury happened. What is more, we labeled the MenSCs with the luciferase gene to better detect the cells in vivo. With a live imaging system, we found that MenSCs could be efficiently delivered to and retained in the injured tissues than in the normal lungs, which was consistent with the results of other reports [12,16,38]. But the retentions between two groups were not statistically significant. This may be caused by individual differences between different mice or the immune system between human and mice. Further studies are needed.

There have been reports that the administration of MSCs could improve both survival and lung inflammation upon in an LPS-mediated ALI animal model [12,39] and in experimental models of bleomycin-induced lung injury [40,41]. In this study, we found that both the inflammatory cells and total protein in BALF were decreased after MenSC administration, while the lung dry/wet ratio was increased. The expressions of VE-cadherin, β-catenin were also upregulated after MenSC administration. Both VE-cadherin and β-catenin are required for maintaining a restrictive endothelial barrier. The higher expressions of VE-cadherin and β-catenin in the lung tissue from mice that received MenSCs suggest that MenSCs could improve pulmonary microvascular permeability.

This demonstrates the restoration of alveolar-capillary membrane function. Most importantly, the MenSC therapy attenuated the observed histopathological impairments and lung texture. These results indicate the potential advantages of MenSCs in repairing lung injury.

Although beneficial effects of the MenSC administration were observed, the precise mechanisms remain unclear. Differentiation, cell–cell contact and paracrine function have all been implicated as possible mechanisms. In this study, we induced the differentiation of MenSCs into epithelial-like cells expressing Surfactant protein B (SPB), Surfactant protein C (SPC) and Surfactant protein D (SPD), indicating the potential application of MenSCs in ALI. However, some studies have suggested that MSC differentiation may not be the main contributor because of poor MSC engraftment and survival at the site of lung injury, as well as the poor possibility of differentiation in a short time [42,43]. Cell–cell contact is also an important factor of cell-based therapy. Studies have suggested that cell–cell contact may enhance the protective effect of MSCs in the injury environment through activation of the programmed death 1 pathway or inhibit naive and memory T-cell responses to their cognate antigens [44,45,46]. Islam et al. also demonstrated Cx43-dependent alveolar attachment and mitochondrial transfer may play an important role in the protection of injured lung [47]. On the other hand, paracrine function has been proposed as the predominant mechanism accounting for the beneficial effects in injury repair [48,49,50]. IL-10 is an anti-inflammatory cytokine secreted by monocytes and play an important role in the downregulates the expression of Th1 cytokines, costimulatory molecules on macrophages and MHC class II antigens. IL-1β is one of the major inflammatory cytokines in ALI/acute respiratory distress syndrome (ARDS) [51]. In this study, with MenSC transplantation, the levels of inflammatory cells and IL-1β expression were significantly reduced, both in the lung tissue and in the BALF, while the expression of the expression of cytokine IL-10 was significantly increased. Since the strong correlation between the number of intravascular neutrophils and MPO has been described [52], the decrease of MPO activities indicated the attenuation of the inflammation. We hypothesized that MenSCs may inhibit function of T cell through cell contact, thereby inhibiting inflammation in injury lung. And the soluble factors of MenSCs may enhance the effect. Further work is needed to confirm this hypothesis. However, a study by Schweitzer et al. [53] confirmed that while AD-MSCs in mice could attenuate lung and systemic injuries via paracrine factors, the effect could not be directly extrapolated to complex animal models. This result suggested that more studies are needed to determine the precise mechanism by which paracrine cytokines contribute to ALI repair.

KGF is an important growth factor in the repair of damaged lung. The benefit of KGF in the injury lung has been confirmed [54,55]. It could promote the proliferation of AT2 and inhibit the apoptosis in the injury lung [56,57]. The higher expression of KGF in the MenSC group demonstrated the effect of MenSCs. In this study, we found the expression of p-gsk3β, p-Src and p-β-catenin were upregulated in the LPS group and downregulated after MenSCs administration while the expressions of PI3K and β-catenin were opposite. The activation of PI3K could induce Akt-dependent phosphorylation and inactivation of gsk3β which may promote the expression of β-catenin [58]. We also found the expression of p-Src, which had been proved to induce the degradation of β-catenin, was decreased after MenSCs implanted. Moreover, an increase expression of PCNA and a decreasing of caspase-3 were also observed. Based on the results above, we assume MenSCs promote the repair of ALI through PI3K/Akt cross-talked with the gsk3β/β-catenin signaling pathway and Src may be involved in the process.

The current study has several limitations. First, we induced ALI by LPS in C57 mice. This mouse model of ALI is focused on inflammation and cannot fully reflect the complexity of clinical ALI/ARDS in human patients. Furthermore, in this study, we only administered the MenSCs intravenously, and we sacrificed the mice 48 h later, which may not fully reflect the clinical application of such a therapy. Gupta et al. [12] did not find significant improvement in ALI in mice after MSC transplantation, although positive results after MSC administration had been previously obtained elsewhere. The dose of LPS, the quantity and sources of MSCs, and the timing and route of MenSC administration may be related to the different results. Studies have confirmed that systemic intravenous MSC delivery may have an efficacy equal to that of direct intratracheal delivery, while that of intraperitoneal delivery is reduced compared with the intratrachealor intravenous delivery routes [5,59,60].

## 4. Materials and Methods

### 4.1. Isolation and Culture of Cells

Menstrual blood were collected and isolated as previously described [18,23]. The donor provided consent for the experiments and written informed consent was obtained from all subjects. The experiments were in accordance with the guidance of “Administration of experimental animals of Zhejiang province” and approved by the Ethics Committee of the First Affiliated Hospital, College of Medicine, Zhejiang University, China (reference number: 2016-290). Briefly, menstrual blood samples were collected with a Divacup (Kitchener, ON, Canada) from healthy women during menstruation and then separated by a density gradient centrifugation with Ficoll-Paque (Fisher Scientific, Waltham, MA, USA) for the mononuclear cells. The interlayer cells were collected and cultured with the Chang Medium (S-Evans Biosciences, Hangzhou, China) in a tissue culture flask (Corning, Corning, NY, USA) for adherent cells. BEAS-2B cells were purchased from the China Centre for Type Culture Collection and cultured in Basal Medium Eagle’s Medium (BMEM) (Lonza, Basel, Switzerland). The cells were cultured in a tissue culture flask until reaching approximately 7–80% confluence. The medium was changed twice a week. The MenSCs used in the experiments were from passages 5 to 8. 

### 4.2. Characterization of the MenSCs

The MenSCs were phenotypically characterized using an FC500 flow cytometer (Beckman, Brea, CA, USA). In brief, MenSCs were digested and resuspended in the staining buffer at a concentration of 1 × 10^6^ cells/mL. Antibodies (10 μL) to cell surface markers including CD29, CD34, CD45, CD73, CD90, CD105, CD117-PE and isotype control (BD, Franklin Lakes, NJ, USA) were added to the buffer, which was then incubated in the dark for 15 min. The cells were then analyzed by flow cytometry as soon as possible.

### 4.3. MenSC Migration

The surface markers of MenSCs including CD140b, CD184, CD185, CD191, CD192, CD194, CD197, CD202b and CD221 (Miltenyi, Teterow, Germany) were detected by flow cytometry.

MenSCs (3 × 10^4^/mL in 100 µL) were seeded on 24-well inserts and BEAS-2B cells were seeded in the 24-well culture plate wells. After the cells became adherent, the medium was replaced with α-MEM without fetal bovine serum (FBS). At a final concentration of 100 ng/mL, LPS was added to the basal chambers of the migration group. Phosphate-buffered saline (PBS) was served as a negative control, and α-MEM with 30% FBS served as a positive control. After 24 h, the cells were stained with Dil for observation by fluorescence microscope.

C57 mice were divided into a control group and an ALI group and anesthetized. LPS at a dosage of 5 mg/kg and the same volume of PBS were instilled intratracheally into the lungs of the migration group and the control group. After 4 h, 1 × 10^6^ MenSCs labeled with the luciferase gene were injected into the mice tail vein and the mice were observed using a live imaging system for 3 days.

### 4.4. Co-Culture of MenSCs and BEAS-2B Cells

MenSCs and BEAS-2B cells were seeded on the transwell inserts and in the culture plate wells at a ratio of 1:5. After the cells became adherent, the inserts were transferred into the wells with BEAS-2B cells, and LPS was added to the basal chambers at a final concentration of 100 ng/mL. BEAS-2B with or without LPS and MenSC were co-cultured for 24 h and then analyzed using a CCK-8 assay (Beyotime Biotechnology, Shanghai, China) to detect the viability, while apoptosis was detected with Annexin V/PI (Thermo Fisher Scientific, Waltham, MA, USA) and a flow cytometer. The viabilities were calculated: viability = (OD_control_ − OD_experiment_)/OD_control_.

### 4.5. MenSC Differentiation into Lung Epithelial Cells

MenSCs were seeded in transwell inserts in a 12-well plate at a volume of 200 µL, and 1 mL of MSC complete medium was added to the basal chamber. After 24 h, the culture medium in the basal chamber was replaced with BMEM, and no medium was added to the insert to induce MenSC differentiation. The medium was changed every 2–3 days. After 2 weeks, the medium was discarded, and the cells were fixed with 4% paraformaldehyde. Normal MenSCs served as a negative control, and BEAS-2B cells served as a positive control. Then, the cells were observed by microscopy or stained with surfactant protein (SP)B, SPC, and SPD antibodies.

MenSC samples were collected before and after differentiation for total RNA extraction. Reverse transcription (RT)-PCR was performed using a QuantScript RT Kit (Tiangen, Beijing, China). PCR was conducted using Taq polymerase (Takara, Shiga, Japan) and a PCR instrument (Thermal) according to the manufacturer’s instructions. The cycling conditions were performed as follows: after an initial denaturation at 95 °C for 15 min, the samples were subjected to 40 cycles at 95 °C for 30 s, 57 °C for 30 s and 72 °C for 1 min, followed by a final step of 72 °C for 10 min. PCR products were resolved on 1% agarose gel stained with ethidium bromide and were visualized using a UV transilluminator (Tanon, Shanghai, China). The primers used in the experiments are described in Table 1, and the reactions were run in triplicate.

### 4.6. Animal Model of LPS-Induced ALI

Male 6 to 8-week-old C57 BL/6J mice purchased from the Shanghai Sippr-BK Laboratory Animal Corporation (Shanghai, China) were anesthetized with 1% pentobarbital sodium (100 mg/mL). ALI was induced with LPS from Escherichia coli O55:B5 (Sigma) at a dosage of 8 mg/kg administered intratracheally, as previously described [12]. After 4 h, PBS (model group) and MenSCs (MenSC group) (1 × 10^6^ cells, 500 μL total volume) were slowly injected into the mice via the tail vein, and normal mice were served as the control group. The mice were examined for X-ray and sacrificed 48 h after. Blood samples were collected and centrifuged at 2000× *g* to obtain plasma samples. The left lung was excised and weighed immediately to determine the wet weight. Then, the lung tissue was placed in an oven at 55 °C for 24 h to determine the dry weight. The dry/wet ratio was defined as dry weight/wet weight. The right lung was fixed with 4% paraformaldehyde to prepare paraffin-embedded sections and stained with Hematoxylin and Eosin (H&E). Immunochemistry was used to detect the changes in proliferating cell nuclear antigen (PCNA) and caspase-3 expression. Another set of mice was then sacrificed. These lungs were washed with 1 mL of PBS twice; the bronchoalveolar lavage fluid (BALF) was collected and centrifuged at 500× *g* for 10 min at 4 °C. The supernatant was stored at −20 °C until testing, and the cell pellet was resuspended in 200 μL for cell counting and cell smear generation.

### 4.7. Detection of BALF Protein and Myeloperoxidase (MPO) Activity

The total protein in the BALF was detected using a BCA kit (Beyotime Biotechnology, Shanghai, China), and the MPO activity was assessed using an MPO kit (Nanjing Jiancheng, Nanjing, China). The IL-1β and IL-10 levels were measured by ELISA (RayBiotech, Norcross, GA, USA) according to the manufacturer’s protocol.

### 4.8. Real-Time PCR Analysis of Cytokines

Total RNA was extracted using TRIzol (Thermo Fisher Scientific, Waltham, MA, USA) and reverse transcriptase. Real-time PCR was conducted using a CFX96 CFX-Touch real-time PCR detection system (Bio-Rad, Hercules, CA, USA). The primers used in the experiments were described in Table 1 and gene β-actin was used as the control. The cycling conditions were as follows: 95 °C for 30 s, 40 cycles of 95 °C for 5 s and 60 °C for 30 s. The reactions were run in triplicate, and the data were analyzed using the 2^−ΔΔ*C*t^ method.

### 4.9. Immunohistochemistry of PCNA and Caspase-3

After de-paraflinization and heated (95 °C) in the 0.01 M sodium citrate buffer (pH 6.0) for 15 min, the paraffin-embedded lung sections were incubated overnight at 4 °C with mouse anti-PCNA and anti-caspase-3 antibodies (CST, Danvers, MA, USA). 3,3′-diaminobenzidine (DAB) was used as the chromogenic substrate and the sections were observed under the microscopy.

### 4.10. Western Blot Analysis

Protein levels of the lungs were measured by western blot. The frozen right lungs were mechanically disrupted and the total protein lysates were extracted using protein lysates (CST). Equal amounts of protein were separated by SDS/PAGE and transferred to polyvinylidene difluoride (PVDF) membranes (Millipore, Bedford, MA, USA). Following blocking with 5% bovine serum albumin for 1h at room temperature, the membranes were incubated at 4 °C overnight with a primary antibody recognizing VE-cadherin (HuaAn, Hangzhou, China), β-catenin (HuaAn), phosphorylated-β-catenin (Thr 41/Ser 45) (HuaAn), Src (JF0947) (HuaAn), phosphorylated—Src (Y529) (Abcam, Cambridge, UK), gsk3β (HuaAn), phosphorylated-gsk3β (Y216 + Y279) (Abcam), PI3K (HuaAn) and β-actin (HuaAn). Then the membranes were incubated with secondary antibodies coupled to horseradish peroxidase at room temperature for 1 h and detected using a Tanon-4500 gel imaging system.

### 4.11. Statistical Analysis

The data were presented as the mean ± standard deviation. The Mann–Whitney U test and ANOVA were applied for the data analyses. *P*-values < 0.05 were considered to represent significant differences.

## 5. Conclusions

MenSCs are pluripotent cells that can be isolated from menstrual blood and can be used to treat many diseases, including ALI. MenSCs could migrate to the lung and attenuate the inflammation of LPS-induced ALI, promoting the repair of the damaged lung tissue. These findings suggest that MSCs have therapeutic potential in ALI, and may be a new therapeutic strategy for LPS-induced ALI.

## Figures and Tables

**Table 1:** Primers Used for Real-Time PCR.

Primer Name	Sequence (5′–3′)	Species
β-actin-Forward	GATGACCCAGATCATGTTTGA	Mouse
β-actin-Reverse	GGAGAGCATAGCCCTCGTAG	
IL-6-Forward	GGCGGATCGGATGTTGTGAT	Mouse
IL-6-R	GGACCCCAGACAATCGGTTG	
IL-1β-Forward	GACCTTCCAGGATGAGGACA	Mouse
IL-1β-Reverse	AGCTCATATGGGTCCGACAG	
TGF-β1-Forward	AATACGTCAGACATTCGGGAAGCA	Mouse
TGF-β1-Reverse	GTCAATGTACAGCTGCCGCACACA	
IL-10-Forward	CACTGCTATGTTGCCTGCTC	Mouse
IL-10-Reverse	TTCATGGCCTTGTAGACACC	
Caspase-3-Forward	TACCGGTGGAGGCTGACT	Mouse
Caspase-3-Reverse	GCTGCAAAGGGACTGGAT	
KGF-Forward	TGGTACCTGAGGATTGACAAACGA	Mouse
KGF-Reverse	CCTTTGATTGCCACAATTCCAAC	
PCNA-Forward	TTGCACGTATATGCCGAGACC	Mouse
PCNA-Reverse	GGTGAACAGGCTCATTCATCTCT	
